# COVID-19 pneumonia in a patient with granulomatosis with polyangiitis on rituximab: case-based review

**DOI:** 10.1007/s00296-021-04905-4

**Published:** 2021-06-06

**Authors:** Alicia Rodriguez-Pla, Holenarasipur R. Vikram, Vanood Khalid, Lewis J. Wesselius

**Affiliations:** 1 Division of Rheumatology, Mayo Clinic Arizona, Scottsdale, AZ USA; 2 Division of Infectious Diseases, Mayo Clinic Arizona, Phoenix, AZ USA; 3 Division of Hospital Internal Medicine, Mayo Clinic Arizona, Phoenix, AZ USA; 4 Division of Pulmonary and Critical Care Medicine, Mayo Clinic Arizona, Phoenix, AZ USA

**Keywords:** COVID-19, SARS-CoV-2, Rituximab, Immunosuppressive treatment, Immunosuppression, B cell depletion, Autoimmune rheumatic disease, Granulomatosis with polyangiitis, GPA, Vasculitis

## Abstract

A 77-year-old man with past medical history of granulomatosis with polyangiitis (GPA) on rituximab and prednisone, presented to the hospital with worsening cough and shortness of breath. He had tested positive for severe acute respiratory syndrome coronavirus 2 (SARS-CoV-2) infection by nasal swab polymerase chain reaction (PCR) while asymptomatic, 6 weeks earlier. He started with cough and shortness of breath 2 weeks after his initial positive test. After developing symptoms, he tested negative twice by nasal swab PCR, but the PCR of his bronchioloalveolar lavage was positive for SARS-CoV-2. He did not develop antibodies against coronavirus. Prednisone 15 mg daily was continued, and he received remdesivir, and convalescent plasma with quick recovery. We reviewed the literature to search for similar cases. Our case suggests that SARS-CoV-2 infection in patients on rituximab may have an atypical presentation and the diagnosis may be delayed due to negative PCR testing in the nasal swab. Patients may benefit from treatment with convalescent plasma.

## Introduction

The extent to which immunosuppressive therapy poses a risk for severe acute respiratory syndrome coronavirus 2 (SARS-CoV-2) infection or coronavirus disease-19 (COVID-19) is still debatable [1]. Due to the development of cytokine storm syndrome in severe COVID-19 cases, steroids and certain immunomodulatory and immunosuppressive medications have been postulated to assist in reversal and recovery [2]. The safety of rituximab in patients with COVID-19 is unclear, and controversy exists whether patients treated with rituximab present with less severe or more profound manifestations [3]. Treatment with a monoclonal antibody cocktail or high-titer convalescent plasma may be beneficial in non-hospitalized and hospitalized non-intubated patients, respectively [4, 5]. We present a patient with a past medical history of granulomatosis with polyangiitis (GPA) on prednisone and rituximab, who initially tested positive for COVID-19 while asymptomatic. He later presented with cough and shortness of breath with negative repeat testing for COVID-19, posing a diagnostic dilemma. We conducted an extensive review of the literature for patients with autoimmune rheumatic diseases on rituximab who presented with COVID-19 infection. Our case resembles and mirrors those reporting an atypical and delayed course of COVID-19 infection in patients on rituximab.

## Case presentation

A 77-year-old man with past medical history of GPA on combination therapy with prednisone and rituximab, presented to our Emergency Department (ED) with cough and shortness of breath.

The patient had been diagnosed with GPA in 2003 when he presented with pulmonary infiltrates, recurrent uveitis and polyarthralgia. He was initially treated with methotrexate and prednisone which controlled his joint symptoms but not uveitis. Between 2006 and 2015, he received a total of six rituximab treatments administered when he had recurrent uveitis. His GPA went into clinical remission for years and rituximab was discontinued. In the spring of 2020, he presented to his outside rheumatologist with several episodes of transient expressive aphasia and leg weakness and was diagnosed with GPA-related pachymeningitis. He was prescribed levetiracetam and prednisone 40 mg po daily, which led to resolution of leg weakness. His rheumatologist recommended resuming rituximab, but patient declined as he was relocating to Arizona.

He was subsequently evaluated in our rheumatology clinic in late July 2020. He was feeling well without leg weakness, speech issues, uveitis, nasal or sinus congestion, cough or shortness of breath, or arthralgia. His complete blood count (CBC) with differential and comprehensive metabolic panel (CMP) were within normal limits, erythrocyte sedimentation rate (ESR) was 2, and C-reactive protein (CRP) of < 3. Additional studies indicated a cytoplasmic-anti-neutrophil cytoplasm antibody (cANCA) of 1:256 with anti-proteinase-3 (PR3)-ANCA was > 8 (negative < 0.4). Perinuclear-ANCA (pANCA) and anti-myeloperoxidase (MPO)-ANCA were both negative. Immunoglobulin (Ig)A: 299 (50–400 mg/dL), IgM: 122 mg/dL (37–286 mg/dL), IgG: 648 mg/dL (767–1590 mg/dL). His urinalysis revealed no blood or protein. His chest X-ray was unremarkable.

He was receiving prednisone 40 mg po daily without concurrent *Pneumocystis jiroveci* pneumonia (PJP) prophylaxis because of allergy to trimethoprim-sulfamethoxazole and dapsone. We recommended continuing prednisone at the same dose of 40 mg po daily and initiated rituximab 1000 mg × 2 IV 2 weeks apart, and atovaquone. After receiving rituximab end of August and in early September, he initiated prednisone taper. Immunoglobulin levels a week after the second infusion of rituximab were: IgA: 261 mg/dL, IgM: 21 mg/dL, IgG: IgG: 573 mg/dL. His cANCA was 1:32 and his ANCA-PR3 antibody was still > 8 (negative < 0.4). CD20: 0, CD19: 0.

The patient tested positive for SARS-CoV-2 infection by nasopharyngeal (NP) swab polymerase chain reaction (PCR) in early October 2020 when visiting out of state family. His only manifestation was fatigue. Two weeks later, he developed a nonproductive cough. Repeat testing for COVID-19 by NP swab PCR at the end of October was negative. The following week, his cough worsened, and he developed shortness of breath. Here, we sent to an outside ED where he was diagnosed with mild pneumonia. He received ceftriaxone and was prescribed azithromycin. Cough and shortness of breath continued to worsen, and he presented to our ED 3 days later.

On arrival to the ED, he was febrile to 38.6 °C, oxygen saturation was 90% on room air and he met sepsis criteria due to tachypnea and fever. Influenza A/B, COVID rapid test, and SARS CoV-2 NP swab PCR, were all negative. He was hospitalized and initiated on intravenous (IV) vancomycin and piperacillin-tazobactam. His other risk factors for adverse outcomes from COVID-19 infection included hyperlipidemia, and coronary artery disease.

His laboratory results on admission revealed: Hemoglobin: 10.9 g/dL, white blood cell count: 5.4 × 10^9^/L, platelet: 211 × 10^3^/L. His CMP was within normal limits. CRP: 72.8 mg/L. c-ANCA: positive at 1:8. ANCA-PR3: 1.2 (negative < 0.4). Coccidioides IgM and IgG EIA, antibody, complement fixation, IgM and IgG by immunodiffusion: negative. MRSA screen nasal: negative: urinalysis: no blood, no protein, no bacteria: D-dimer was elevated at 1570 (normal <  = 500 ng/mL FEU.)

**Fig. 1:**
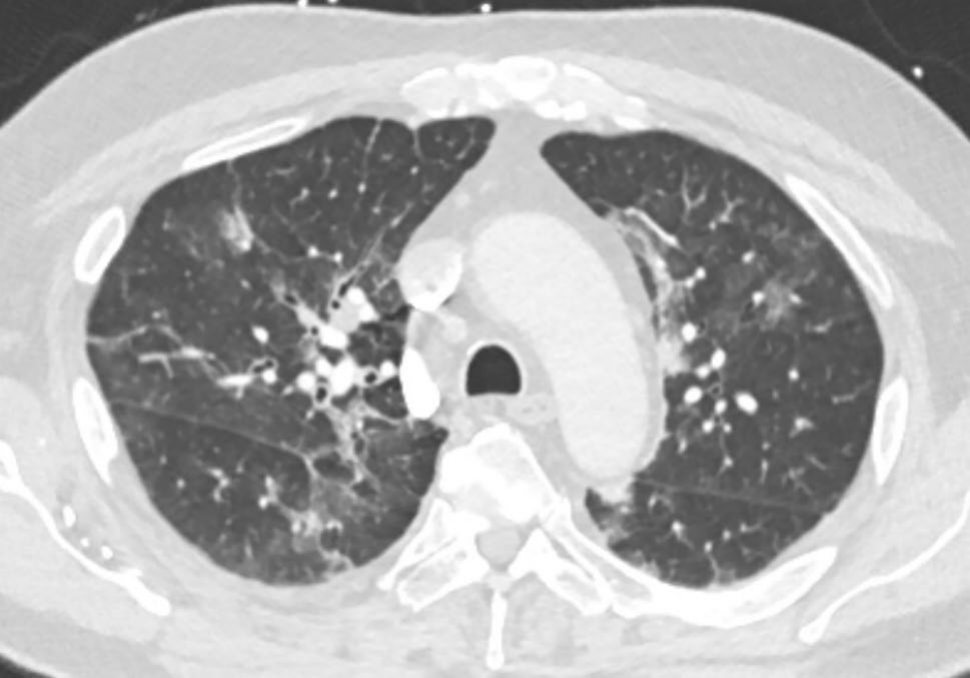
Computerized tomography of the chest revealed multifocal groundglass opacities throughout both lungs, predominantly surrounding vessels and most prominent in the right upper lobe with some foci of groundglass consolidation or more veins with some associated septal line thickening. The findings were concerning for multifocal hemorrhage related to acute exacerbation of GPA versus viral infection

A chest X-ray revealed patchy airspace densities in the left mid to lower lung, right lower lung, right upper lobe, likely representing pneumonia. A chest CT angiogram was negative for pulmonary embolism, but revealed multifocal groundglass opacities throughout both lungs, predominantly surrounding vessels and most prominent in the right upper lobe. There was associated septal line thickening, concerning for multifocal hemorrhage secondary to an exacerbation of GPA versus viral infection (Fig. 1).

He had previously tested positive for COVID via NP swab on 10/02/2020 but was subsequently negative on 10/26/2020 and 11/15/2020. A bronchoscopy was performed with bronchioalveolar lavage (BAL) indicating ongoing inflammation with 22% neutrophils noted in the lavage cell differential. Cytology was negative for malignancy and no fungal organisms or viral inclusions were identified. BAL SARS CoV-2 PCR returned positive, confirming COVID-19 pneumonia. Extensive additional studies on his BAL (our immunocompromised host panel) for bacterial, fungal, mycobacterial cultures, PJP smear and PCR, aspergillus antigen, legionella PCR and culture, nocardia stain, acid-fast smear, and fungal smears were all completely negative.

Infectious disease consultant recommended treatment with remdesivir for 5 days in the setting of immunosuppression due to prednisone and recent treatment with rituximab, COVID-19 pneumonia, and hypoxia. Prednisone 15 mg po daily was continued. His serum SARS-CoV-2 total antibodies came back negative indicating lack of humoral immune response to SARS-CoV-2 infection. He received two units of convalescent plasma.

The patient’s liver function tests remained within normal limits. His inflammatory markers trended down. He continued to require 2 L of oxygen intermittently and was discharged home on oxygen 6 days after admission. A month later, he tested positive for SARS-CoV-2 nucleocapsid total antibodies, and he discontinued oxygen. Three months later, he was doing well he received the Pfizer COVID-19 vaccine, and the plan was to re-treat him with rituximab only if he had a clinical relapse.

## Search strategy

Using the PubMed/MEDLINE, Scopus, Web of Science and LitCOVID databases, we searched existing literature using the following strategy: (COVID-19 OR SARS-CoV-2 OR coronavirus) AND ((autoimmune diseases) OR (rheumatic diseases) OR (granulomatosis with polyangiitis) OR vasculitis) AND (rituximab OR (biologic therapy)). Only publications involving humans were reviewed. Three-hundred-and-twenty-two publications were retrieved from PubMed/MEDLINe, 171 from Scopus, 58 from Web of Science and 53 from LitCOVID. After excluding non-relevant papers, all remaining cohorts, case series, and case reports published before 05/22/2021 were reviewed.

## Discussion

We report an immunosuppressed patient with GPA who presented with an atypical and progressive SARS-CoV-2 infection with delayed onset. Although we cannot draw definite conclusions from a single patient, our case suggests that rituximab can contribute to worsening COVID-19 infection. Negative repeat NP swab testing with worsening cough and hypoxia indicates that he had COVID-19 pneumonia due to immune suppression. Negative NP swab PCR in patients with COVID-19 lower respiratory infection has been well described.

Traditionally, patients on immunosuppressive therapy including those with autoimmune rheumatic diseases, have been considered to be at an increased risk for infections, including viral infections [6–8]. However, in severe cases of SARS CoV-2 infection, hypoxic respiratory failure and multi-organ failure are thought to be caused by “cytokine storm syndrome”, which is triggered by rapid virus replication eliciting an explosive inflammatory reaction mediated by release of cytokines and chemokines [9, 10]. Therefore, modulation of the immune response or suppression of over-reactive cytokine production may be an effective therapy in severe cases [10].

Initial studies did not support an increase in the risk of respiratory or life-threatening complications from SARSCoV-2 in patients with rheumatic diseases on immunosuppression [11, 12]. Rituximab is a chimeric monoclonal anti-CD20 antibody that has been approved for treatment of several autoimmune rheumatic diseases, including GPA. Rituximab influences B cell cytokine network with a secondary influence on T helper cell response [13]. B cell depletion could jeopardize antiviral immunity by blocking production of SARS-CoV-2 antibodies [3]. The safety of rituximab in the context of COVID-19 is still unclear.

Initial results from the European League Against Rheumatism (EULAR) COVID-19 registry did not suggest worse outcomes in 37 patients who had been treated with rituximab [14]. In a cross-sectional study of 206 patients, 1 out of 77 patients on rituximab tested positive for COVID-19 immediately post-rituximab administration and had an uneventful course despite being elderly [15]. A recent multicenter study from Turkey concluded that biological treatment, including rituximab, does not seem to affect COVID-19 outcomes [16].

The case of a 25-year-old male who developed moderate COVID-19 with fever and mild dyspnea despite having received high-dose steroids, cyclophosphamide, and rituximab for eosinophilic granulomatosis with polyangiitis (EGPA) was reported [17]. Furthermore, some authors have reported mild cases of COVID-19 in patients treated with rituximab [18–20]. A woman with GPA treated with rituximab and low-dose steroids presented with severe symptoms of COVID-19 a few days after she received maintenance rituximab therapy, but her symptoms developed more progressively than in most COVID-19 patients and she eventually recovered. The authors suggested that steroids and rituximab may decrease the cytokine storm associated with COVID-19 and facilitate recovery [18]. A patient with microscopic polyangiitis (MPA) developed COVID-19 infection 3.5 months after rituximab treatment for a relapse and was treated at home with acetaminophen and improved. Her antibodies against SARS-CoV-2 were positive a month after her disease and the authors suggested that B cell depletion may favor a milder course and the generation of antibodies [19]. In another report, it was suggested that the lack of antiviral antibodies might have prevented severe disease in a patient treated with rituximab for GPA who developed a mild case of COVID-19 and who was able to clear the infection even in the absence of serological response to the virus [20].

In contrast, several cohorts of patients with autoimmune rheumatic diseases on immuno-suppressants from Spain were reported to develop severe disease with fatalities after treatment with rituximab [21–23]. In a study of 76 patients on rituximab that were screened, 17.1% had suspected or confirmed SARS-CoV-2 infections and of those, 61.5% were hospitalized due to severe disease, and 3 died. The authors concluded that rituximab should be considered a possible risk factor for unfavorable outcomes in COVID-19 patients with rheumatic diseases [23]. Hassali et al. reported that 67% of the patients treated with rituximab in the National Registry for patients with rheumatic diseases infected with SARS-CoV-2 in Germany required hospitalization [24]. Two recent European studies reported that rituximab therapy is associated with more severe COVID-19 [25, 26].

In addition, several case reports indicate patients with rheumatic diseases treated with rituximab develop severe SARS-CoV-2 infections. A patient with systemic sclerosis and pulmonary involvement treated with rituximab died after requiring mechanical intubation and receiving treatment with tocilizumab [27]. Schulze-Koops et al. described two fatal outcomes in patients with rheumatoid arthritis treated with rituximab [28]. Benucci et al. reported a case of severe COVID-19 in a patient with myositis treated with rituximab, who eventually recovered after invasive ventilation and treatment with remdesivir, tocilizumab, dexamethasone, convalescent plasma and intravenous immunoglobulins, who had absence of antibodies to SARS-CoV-2 even up to 4 weeks after discharge [29]. Three patients with systemic sclerosis and several patients with GPA have been reported to develop atypical late clinical worsening similar to the clinical course in the patient we report [18, 30]. A case of persistent viral shedding with no cytokine storm in a patient with GPA on rituximab has also been reported [31]. Hakroush et al. reported a case of delayed COVID-19 diagnosis in a patient with GPA on rituximab due to repeated negative tests as in the case of our patient, whose diagnosis was finally made by a tracheal aspirate and eventually died [32]. A long, relapsing, and atypical symptomatic course of COVID-19 in a B-cell-depleted patient after rituximab treatment for GPA has also been reported [33]. A second COVID-19 infection in a patient with GPA on rituximab has recently been reported [34].

Our patient presented with a case of delayed COVID-19 diagnosis given negative NP testing. We consider that his immunosuppressive treatment with B cell depletion therapy resulting in an impaired serological response, hindered clearance and resolution of the infection, and contributed to the progression of the SARS-CoV-2 infection to the lower respiratory track. Treatment with remdesivir and convalescence plasma helped him to recover.

## Conclusion

Current clinical studies and case reports should caution clinicians about re-treatment of patients with rituximab who are at risk for COVID-19 infections, as these patients may present with atypical features and have increased risk for severe lower respiratory tract disease. The lack of serological response following rituximab may hinder SARS-CoV-2 clearance and resolution of infection. In patients with GPA or other autoimmune disorders that involve the lungs, it is of vital importance to consider and exclude infections including COVID-19, before enhancing immune suppression to treat a possible exacerbation of the underlying rheumatologic disease. It is also important to be aware that in patients with respiratory symptoms and abnormal findings on chest imaging, a negative NP swab SARS-CoV-2 PCR does not exclude COVID-19 pneumonia. Hospitalized patients should be maintained under adequate respiratory precautions until repeat PCRs and lower respiratory tract samples have been tested for COVID. Monoclonal antibody cocktail directed against SARS-CoV-2 should be entertained in symptomatic outpatients with COVID-19 infection, especially in the context of underlying immune suppression. If hospital admission is required, such patients should be considered for early infusion of convalescent plasma in addition to other therapies, such as remdesivir and dexamethasone, depending on the severity of illness and extent of hypoxia.

## Data Availability

Possible.
